# Microstructural White Matter Changes, Not Hippocampal Atrophy, Detect Early Amnestic Mild Cognitive Impairment

**DOI:** 10.1371/journal.pone.0058887

**Published:** 2013-03-14

**Authors:** Lin Zhuang, Perminder S. Sachdev, Julian N. Trollor, Simone Reppermund, Nicole A. Kochan, Henry Brodaty, Wei Wen

**Affiliations:** 1 Centre for Healthy Brain Ageing, University of New South Wales, Randwick, New South Wales, Australia; 2 Neuropsychiatric Institute, Euroa Centre, Prince of Wales Hospital, Randwick, New South Wales, Australia; 3 Primary Dementia Collaborative Research Centre, School of Psychiatry, Faculty of Medicine, University of New South Wales, Randwick, New South Wales, Australia; 4 Department of Developmental Disability Neuropsychiatry, School of Psychiatry, Faculty of Medicine, University of New South Wales, Randwick, New South Wales, Australia; University of Maryland, College Park, United States of America

## Abstract

**Background:**

Alzheimer’s disease (AD) is generally considered to be characterized by pathology in gray matter of the brain, but convergent evidence suggests that white matter degradation also plays a vital role in its pathogenesis. The evolution of white matter deterioration and its relationship with gray matter atrophy remains elusive in amnestic mild cognitive impairment (aMCI), a prodromal stage of AD.

**Methods:**

We studied 155 cognitively normal (CN) and 27 ‘late’ aMCI individuals with stable diagnosis over 2 years, and 39 ‘early’ aMCI individuals who had converted from CN to aMCI at 2-year follow up. Diffusion tensor imaging (DTI) tractography was used to reconstruct six white matter tracts three limbic tracts critical for episodic memory function - the fornix, the parahippocampal cingulum, and the uncinate fasciculus; two cortico-cortical association fiber tracts - superior longitudinal fasciculus and inferior longitudinal fasciculus; and one projection fiber tract - corticospinal tract. Microstructural integrity as measured by fractional anisotropy (FA), mean diffusivity (MD), radial diffusivity (RD) and axial diffusivity (AxD) was assessed for these tracts.

**Results:**

Compared with CN, late aMCI had lower white matter integrity in the fornix, the parahippocampal cingulum, and the uncinate fasciculus, while early aMCI showed white matter damage in the fornix. In addition, fornical measures were correlated with hippocampal atrophy in late aMCI, whereas abnormality of the fornix in early aMCI occurred in the absence of hippocampal atrophy and did not correlate with hippocampal volumes.

**Conclusions:**

Limbic white matter tracts are preferentially affected in the early stages of cognitive dysfunction. Microstructural degradation of the fornix preceding hippocampal atrophy may serve as a novel imaging marker for aMCI at an early stage.

## Introduction

As Alzheimer’s disease (AD) is a progressive neurological condition, identifying changes early in the disease process is of clinical importance to enable early diagnosis and intervention. The construct of mild cognitive impairment (MCI) captures the earliest clinical features of dementia, and may be an appropriate stage for clinical enquiry [1], [2]. Previous clinic- and population-based studies have reported that individuals with amnestic MCI (aMCI) are at a manifold increased risk of progressing to dementia, in particular AD [3], [4], [5], [6], [7], [8]. Post-mortem studies have found AD neuropathologic hallmarks in the brains of aMCI patients, and recent imaging studies of amyloid deposition using positron emission tomography have shown that aMCI individuals are likely to have an increased brain load of amyloid, and this is related to progression to AD [9], [10].

Gray matter (GM) atrophy is a prominent feature of aMCI, with the hippocampus being the earliest and most vulnerable region to become affected [11], [12], [13], [14], [15]. Despite the majority of research in MCI focusing on GM, a growing number of evidence from post-mortem studies [16], [17] has revealed that the brains of dementia patients also exhibit white matter (WM) pathology, such as axonal loss, demyelination, and death of oligodendroglial cells. Traditionally, WM degeneration is considered a secondary pathological event following GM damage [18]. However, recent neuroimaging studies found substantial WM degradation but intact GM structure in subjective MCI [19] and aMCI [20], even in cognitively normal elderly destined to develop aMCI [21]. In addition, WM lesions instead of hippocampal atrophy predicts incident AD [22]. Similarly, remarkable WM atrophy was found in the brains of preclinical AD patients at autopsy, whereas cortical GM was still normal [23]. Furthermore, AD animal model studies [24], [25] showed that axonal and myelin injury of WM is present before the formation of amyloid deposition and neurofibrillary tangles in GM. This converging evidence suggests that WM degradation may play an important role in the pathogenesis of cognitive impairment and even lie upstream of GM loss in the earlier stage of cognitive decline.

Previous studies using diffusion tensor imaging (DTI) fiber tractography have found decreased microstructural integrity of major WM tracts in aMCI, but the results varied considerably across these studies. For instance, one study reported compromised fornix WM integrity and unchanged parahippocampal cingulum [26], while others found either abnormalities of the posterior cingulum and intact uncinate fasciculus [27] or no significant WM changes of the fornix, uncinate fasciculus, and cingulum bundle in MCI [28], relative to controls. One explanation for these inconsistent findings is that different disease stages of the heterogeneous MCI population may have various degrees of WM damage. Therefore, further studies examining the development of WM damage at different stages of MCI is warranted. In addition, despite an effort to quantify WM degeneration in previous DTI studies, it is unclear whether WM damage and GM atrophy occurs sequentially or simultaneously.

To examine WM tract degeneration and its relationship with GM atrophy in prodromal stages of AD, this study measured WM integrity and hippocampal volumes at two stages of aMCI – those who recently converted to aMCI from normal cognition previously, and those who had received the diagnosis of aMCI for at least two years. DTI tractography was used to examine three limbic WM tracts (the fornix, the parahippocampal cingulum, and the uncinate fasciculus), two cortico-cortical association WM tracts (the superior longitudinal fasciculus and the inferior longitudinal fasciculus) and one projection fiber tract comprising the corticospinal tract. Given the functional importance of the three limbic WM tracts in episodic memory [29], [30], [31] and the fact that episodic memory impairment is the earliest and most prominent clinical symptom in the course of AD, it was hypothesized that (i) limbic WM tracts would be preferentially more vulnerable than cortico-cortical association fibers and corticospinal tract in the early stage of the disease process, (ii) primary WM degradation would precede hippocampal atrophy in the earlier stage of aMCI.

## Materials and Methods

### Ethics

Written informed consent was obtained from each participant. The study was approved by the Ethics Committees of the University of New South Wales and the South Eastern Sydney and Illawarra Area Health Service and was in accordance with the Declaration of Helsinki.

### Subjects

All participants were recruited from the Sydney Memory and Ageing Study (MAS), a population-based longitudinal study of non-demented older people aged 70–90 in Sydney, Australia. Participants were excluded if they had a history of dementia, schizophrenia, bipolar disorder, multiple sclerosis, motor neuron disease, developmental disability, progressive malignancy, a Mini-Mental State Examination (MMSE) <24 adjusted for age and education, insufficient English language abilities to complete assessment, or any medical or psychological conditions that might interfere with clinical and neuropsychological assessment.

Participants in the present study, who were assessed at two waves, two years apart, were 155 cognitively normal (CN) individuals (age range, 72.47 to 90.47 yrs) who were cognitively normal at both waves, 27 ‘late’ aMCI subjects (age range, 74.05 to 88.81 yrs) who were cognitively impaired in the memory domain at both waves, and 39 ‘early’ aMCI subjects (age range, 72.95 to 90.72 yrs) who were cognitively normal at wave 1 and diagnosed with aMCI at wave 2. Details of the demographic profiles of all participants are provided in Table 1.

**Table 1:** Demographic and cognitive profiles of the study participants.

	CN	Late aMCI	Early aMCI	F	p value
	Mean ± SD	Mean ± SD	Mean ± SD		
Age (years)	79.08±4.36	81.01±4.60	80.74±5.29	3.506	0.032
Sex (M/F)	61/94	18/9	24/15	11.136	0.004
Education (years)	11.99±3.65	12.28±4.15	13.00±4.37	1.072	0.344
Episodic memory	0.221±0.944	−2.216±0.845	−1.356±1.103	92.236	<0.001^a^ ** ^,b^ ** ^,c^ *
APOE4,+/1	33/122	9/18	7/32	2.422	0.298
WMHs (percent ICV)	0.991±0.948	1.109±0.753	0.927±0.751	0.346	0.708
Left HV mm^3^Right HV mm^3^	3591±4393351±420	3370±5293187±455	3564±5003344±433	4.0752.917	0.018^a^ *0.056

CN, cognitively normal; aMCI, amnestic mild cognitive impairment; WMHs, white matter hyperintensities; HV, hippocampal volume.

a significant difference between late aMCI and cognitively normal controls.

b significant difference between early aMCI and cognitively normal controls.

c significant difference between late aMCI and early aMCI.

* Significance at p<0.05.

** Significance at P<0.001.

Diagnosis of MCI was made based on the recent international consensus criteria [32], adapted as follows: (a) complaint of decline in memory or other cognitive functions from the participant or a knowledgeable informant; (b) generally intact instrumental activities of daily living (IADL), measured by an average score <3.0 on the Bayer ADL Scale adjusted for physical impairment [33]; (c) objective cognitive impairment defined as at least 1.5 SD below the normative data on any one neuropsychological test; (d) MMSE score of ≥24 adjusted for age and education. MCI was further classified into two subtypes: aMCI (presence of memory impairment) and naMCI (no memory impairment but at least one non-memory domain impaired). Combined performance on verbal episodic memory tasks was calculated from the average z-scores of Logical Memory (LM) delayed recall (Story A) [34] and three measures from the Rey Auditory Verbal Learning Test (RAVLT) [35]: total learning sum of trials 1–5 (RAVLTtotr), short-term delayed recall trial 6 (RAVLT6) and long-term delayed recall trial 7 (RAVLT7).

### Magnetic Resonance Imaging Acquisition

The MRI scans used in the current study are from the second wave of the MAS study beginning in Oct 2007 and finishing in Dec 2009. All subjects underwent MRI scanning on a Philips 3T Achieva Quasar Dual MRI scanner (Philips Medical System, Best, The Netherlands) located at Neuroscience Research Australia (NeuRA), Sydney, Australia. For each participant, the MRI scan was obtained within 3 months after the neuropsychological testing. 32 directional DTI data (b = 1000 s/mm^2^) with three no-diffusion weighted b0 images were acquired with a single-shot, spin-echo, echo-planar imaging (EPI) sequence: echo time (TE) = 68 ms, repetition time (TR) = 7767 ms, flip angle = 90°, matrix size = 240×240, field of view (FOV) = 240×240 mm, yielding in-plane resolution of 1×1 mm, 55 2.5 mm contiguous axial slices without gap. Each DTI scan was repeated twice to increase the signal to noise ratio (SNR). High-resolution T1-weighted structural images were acquired for hippocampal volumetry analysis using turbo field echo sequence with the following protocols: TE = 2.61 ms, TR = 5.75 ms, flip angle = 8°, FOV = 256×256×190 mm, 1 mm slice thickness with no gap, voxel size = 1×1×1 mm^3^. T2-FLAIR was acquired with TR = 10000 ms, TE = 110 ms, matrix size = 512×512, slice thickness = 3.5 mm without gap, and in plane resolution = 0.488×0.488 mm.

### Diffusion Tensor Imaging Preprocessing

DTI data were pre-processed and analysed with the FMRIB’s Diffusion Toolbox (FDT) of the FMRIB’s Software Library (FSL) version 4.1.7 (http://www.Fmrib.ox.ac.uk/fsl, Oxford Centre for Functional Magnetic Resonance Imaging of the Brain, Oxford University, UK). First, each subject’s raw DTI data were visually inspected to exclude any severe head movements and DTI artifacts. Second, eddy current correction was performed by linearly registering each diffusion-weighted volume to the reference T-weighted non-diffusion b0 image, using the FMRIB’s linear image registration (FLIRT). A binary brain mask was created to remove the non-brain tissue using the Brain Extraction Tool (BET) in FSL. Then, diffusion tensor model was fitted to each imaging voxel of the pre-processed DTI data in the native diffusion space to derive fractional anisotropy (FA), mean diffusivity (MD), radial diffusivity (RD), and axial diffusivity (AxD) maps.

### Probabilistic Fiber Tractography of WM Tracts

**Figure 1:**
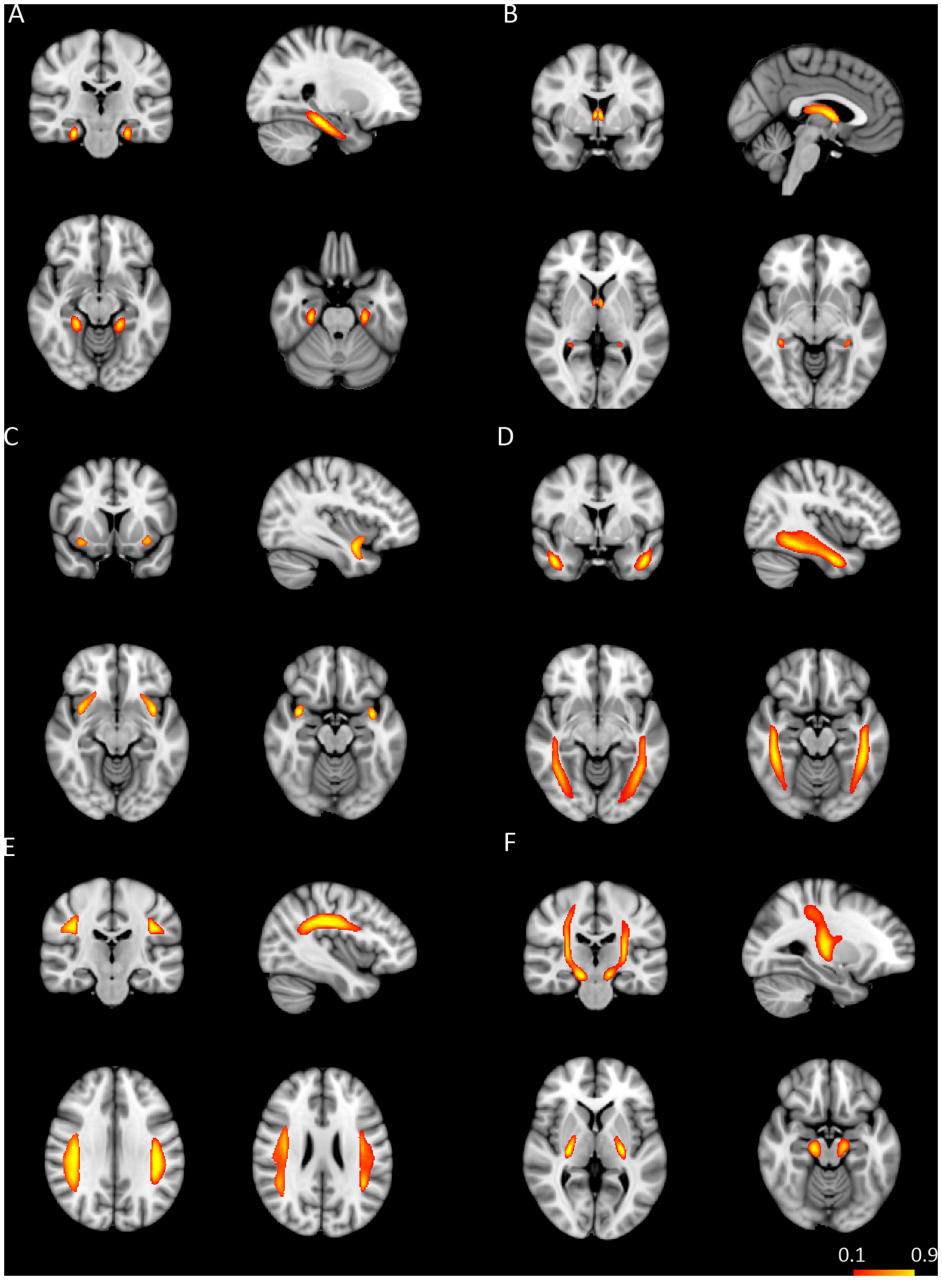
Probabilistic maps of the parahippocampal cingulum (A), the fornix (B), the uncinate fasciculus (C), inferior longitudinal fasciculus (D), the superior longitudinal fasciculus (E), and the corticospinal tract (F). All tracts were superimposed on the MNI T1 template. Only voxels present in at least 10% of the study participants are shown in red-yellow. The colour bar denotes the percentage of subjects in whom the reconstructed fiber tract exists.

**Figure 2:**
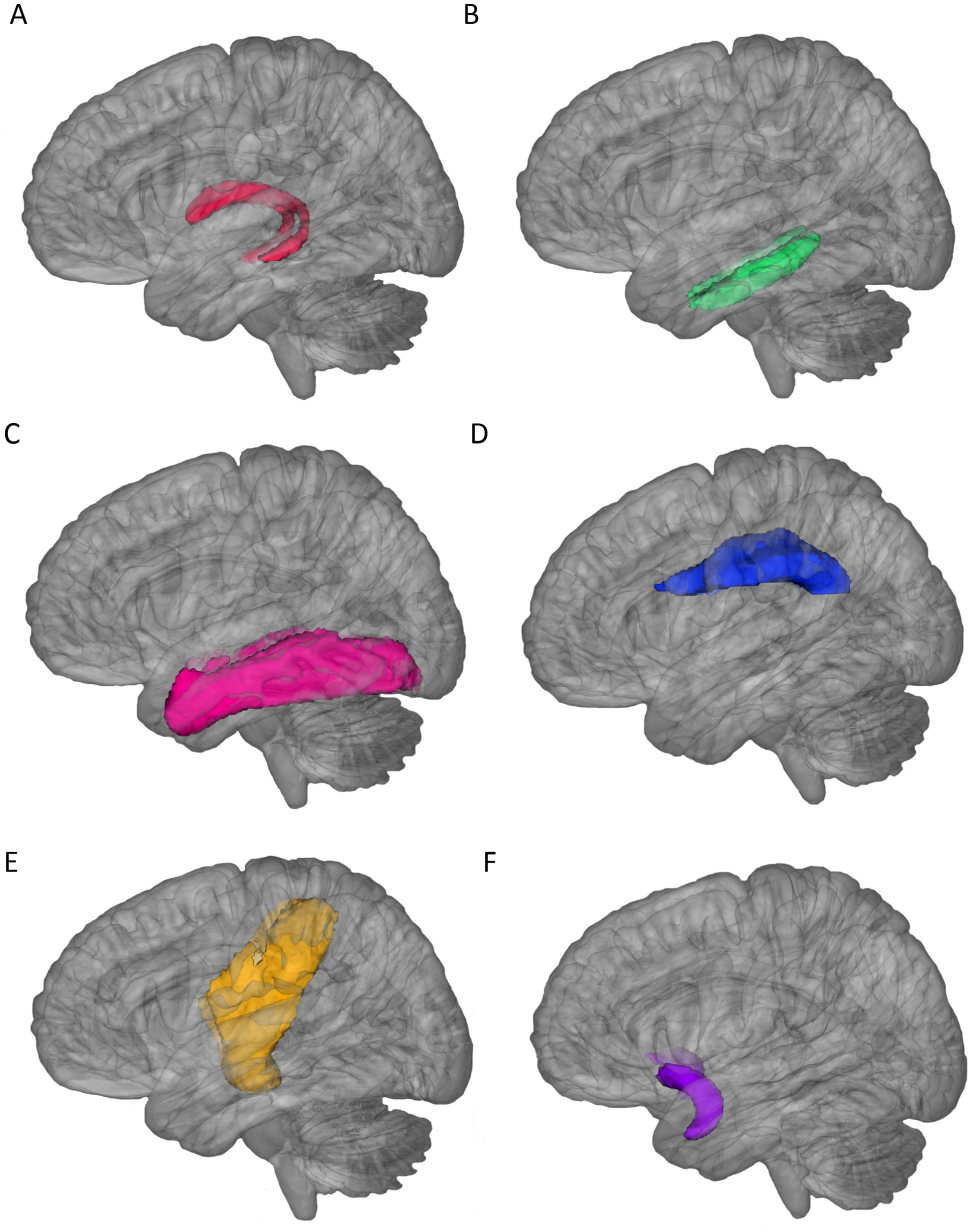
3D rendering of probabilistic maps of the fornix (A), the parahippocampal cingulum (B), the inferior longitudinal fasciculus (C), and the superior longitudinal fasciculus (D), and the corticospinal tract (E), and the uncinate fasciculus (F). All tracts were overlaid on a 3D rendering of the MNI T1 template.

Fiber tractography was performed for each subject in the diffusion space to delineate fiber tracts using a probabilistic crossing-fiber tracking algorithm implemented within the FDT toolbox of the FSL software. Prior to running fiber tracking, Bayesian Estimation of Diffusion Parameters (BEDPOSTX) using Markov Chain Monte Carlo sampling was conducted on the output of eddy-current corrected DTI data to generate distributions of diffusion parameters and model crossing fibers at each image voxel. After applying BEDPOSTX to eddy-current corrected DTI data, fiber tracking was initiated with 5000 streamline samples from all voxels within the seed mask to create a connectivity distribution of each fiber tract in the original diffusion space. The output fiber connectivity map contained a connectivity value at each voxel, representing the number of streamlines projecting from the seed region and passing through the waypoint mask. In order to remove image noise, each fiber connectivity image was then thresholded to include only voxels with a connectivity value of 20% of the maximum connectivity value or more. The thresholded fiber connectivity map was binarized to create a mask of each fiber tract. Then, mean FA and MD values were calculated within the mask of the corresponding fiber tract in the diffusion space. In addition, probability maps of six WM tracts were created to check the anatomical consistency of the same tract across all subjects (Figure 1 & 2). In terms of probability maps of fiber tracts, FLIRT in FSL was first used to co-register each subject’s DTI image to their own T1 image, which simultaneously generates the linear transformation matrix M1. Then, by using FMRIB’s linear image registration (FNIRT) in FSL, each subject’s T1 image was spatially normalized to the MNI space to generate the non-linear transformation matrix M2. By applying the transformation matrix M1 and M2, each subject’s fiber tract in the diffusion space was brought to the MNI standard space. Finally, the spatially normalized fiber tracts of all subjects were averaged to create the probability map of each tract.

### Region of Interest Definition

A two-ROI approach was applied to draw seed and waypoint ROIs on the colour-coded FA map of each subject to isolate specific fiber tracts. These ROIs were manually placed by a single rater (L.Z) blind to clinical information, based on a previously published protocol [36] which has been shown to be highly reliable and reproducible. The ROIs for each fiber tract were defined as follows: (i) *Uncinate fasciculus*: the seed ROI was placed to include temporal lobe WM on contiguous coronal slices, from the most anterior part of the temporal lobe to the point where the temporal lobe was separated from the frontal lobe. On the same coronal slice that the seed mask was terminated, the waypoint ROI was drawn in the frontal WM, which is known to constitute the uncinate fasciculus. (ii) *Inferior longitudinal fasciculus*: the seed ROI was the same as that of the uncinate fasciculus. The waypoint ROI was defined on a single coronal slice immediately posterior to the edge of the splenium of the corpus callosum, to avoid the interference of the uncinate fasciculus projecting from the same seed region. (iii) *Fornix*: the seed mask was drawn on contiguous axial slices at the level of the body of the fornix. The waypoint mask was placed on a single axial slice using the posterior part of the thalamus as the landmark, as the crus of the fornix (barely visible on the colour-code map) is adjacent to this area. (iv) *Corticospinal tract*: the seed ROI was defined on an axial slice on which the cerebral peduncle was visible. The waypoint ROI was delineated on the precentral and postcentral gyrus using the central sulcus as the landmark. (v) *Parahippocampal cingulum*: the seed ROI was placed on three contiguous coronal slices inferior to the splenium of the corpus callosum to delineate the parahippocampal cingulum. The waypoint ROI was placed on a single coronal slice anterior to the pons in the mid-sagittal plane. (vi) *Superior longitudinal fasciculus*: the seed ROI lateral to the corona radiata was drawn on a single coronal slice posterior to the postcentral gyrus. No waypoint ROI was required, as the seed ROI did not contain other fiber tracts.

### White Matter WM Hyperintensities Analysis

**Figure 3:**
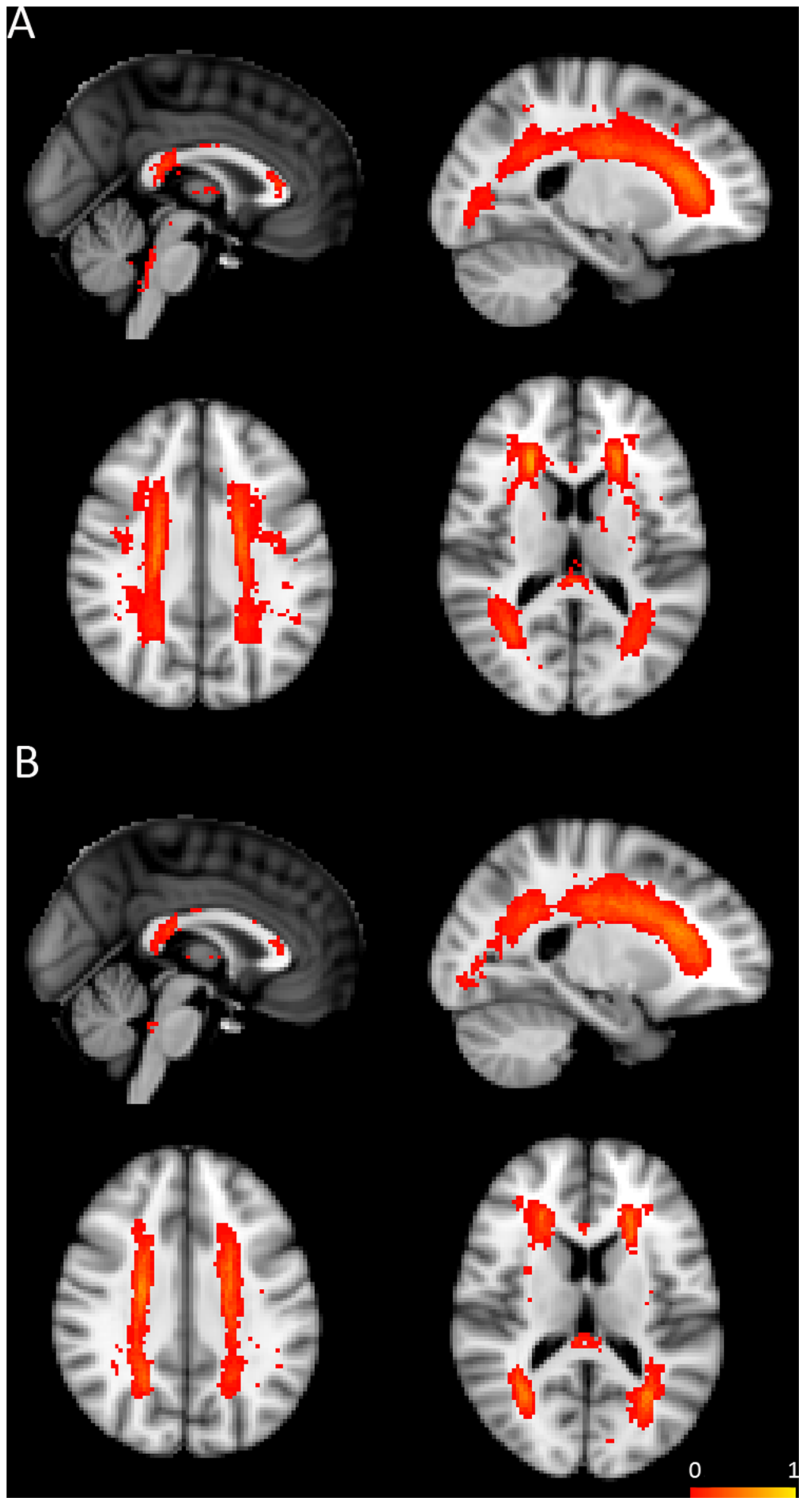
Probability maps of white matter hyperintensities (WMHs) from early (A) and late aMCI (B). WMHs were shown in the red-yellow and superimposed on the MNI T1 template. The color bar denotes the percentage of subjects who had WMHs in each image voxel.

WM hyperintensities (WMHs) for each subject were extracted using our in-house software [37], [38] to investigate whether WMHs have a potential influence on the DTI measures of the WM tracts. Briefly, the automated measurement of the WMHs included the following steps: (1) each subject’s T1-weighted structural images were co-registered into their own T2-FLAIR images; (2) T2-FLAIR and T1 images were spatially normalized to the MNI standard space; (3) Both T1 and T2-FLAIR images were segmented into gray matter, WM and CSF; (4) WMHs from WM regions were automatically detected by the in-house software. A group probability map of WMHs (Figure 3) was generated by averaging the binarized WMHs image of each aMCI subject in the MNI space.

### Volumetric Analysis of the Hippocampus

Automated segmentation of bilateral hippocampi was performed using the FMRIB’s Integrated Registration and Segmentation Tool (FIRST) [39] as implemented in FSL. First, each subject’s T1 image was spatially transformed to the MNI 152 standard space using 12 degrees of freedom affine registration as implemented in FLIRT [40]. The resultant linear transformation matrix was applied to an MNI152 sub-cortical mask to remove voxels outside the sub-cortical regions in each individual. Second, FIRST was run to extract the hippocampus using a Bayesian framework of shape and appearance models reconstructed from a training set consisting of 336 manually segmented T1 images from the Centre for Morphometric Analysis, Massachusetts General Hospital, Boston. For hippocampal segmentation 30 modes of variation was selected. FIRST then searched through linear combinations of shape modes of variation for the most probable shape of the hippocampus for each subject. Third, boundary correction was performed to determine which boundary voxels belonged to the hippocampus. Finally, each subject’s hippocampal size was calculated by multiplying the number of voxels within the hippocampal mask with the voxel size (1 mm^3^). Additionally, intracranial volume (ICV) was calculated as the total volume of GM, WM, and CSF using the segmentation toolbox in SPM (Statistical Parametric Mapping) 5 (Wellcome Trust Centre for Neuroimaging, UK).

### Statistical Analysis

One-way analysis of variance (ANOVA) was performed to test the group difference in continuous variables of demographic and neuropsychological data, and categorical data were compared using Pearson’s Chi-square test. Analyses of covariance (ANCOVAs) with Bonferroni adjustment for multiple comparisons were performed to assess the group difference in DTI, WMHs and hippocampal volumetric measures, while including age, sex, years of education and APOE genotype as covariates of no interest. DTI measures of WM tracts showing significant between group differences were then entered into a series of stepwise linear regression models as dependent variables, while including WMHs, hippocampal volume, age, sex, years of education, and APOE genotype as independent variables to determine which variable was most predictive of WM changes in early and late aMCI. All statistical analyses were performed using the PASW software package version 18 (SPSS, Inc., Chicago, IL, USA).

## Results

### Demographic and Cognitive Characteristics

As shown in Table 1, there were significant differences in age, sex, and performance on episodic memory tasks. Post-hoc analysis revealed that compared with cognitively normal controls, both early and late aMCI subjects had poorer performance on the tasks of verbal episodic memory. Late aMCI individuals were more impaired in episodic memory functioning than early aMCI individuals. In comparison with controls, there was a greater proportion of male both aMCI groups. The prevalence of APOE4 carriers and years of education did not differ among the three groups.

### WMHs and Hippocampal Volumetric Analysis

After correcting for age, sex, years of education and ICV, there were no significant differences among the three diagnostic groups in total WMHs burden. Left hippocampal volumes were significantly different among the three groups whereas right hippocampal volumes were not. Post-hoc analysis showed that the left hippocampus was significantly atrophic in late aMCI subjects (p = 0.016) compared with controls. No significant differences in the hippocampal volume were found between early and late aMCI, or between early aMCI and controls.

### Group Comparisons of DTI Measures of Fiber Tracts

FA and MD values of the three groups are shown in Table 2. Among the three groups, significant FA value differences were observed in the bilateral fornices and the left uncinate fasciculus, and MD values were significantly different in the left parahippocampal cingulum, bilateral fornices, and bilateral uncinate fasciculus. Post-hoc comparisons were also carried out with the following results: (i) compared with controls, late aMCI subjects showed significantly lower FA values in the left fornix while MD values were significantly greater in the left parahippocampal cingulum, fornices bilaterally, and uncinate fasciculus bilaterally; (ii) compared with controls, early aMCI subjects had significant MD increases in the bilateral fornices without any reduction in FA values, (iii) between the two aMCI groups, late aMCI subjects had significantly greater MD in the bilateral uncinate fasciculus compared with early aMCI. The repeated measures ANCOVA did not reveal any significant hemisphere effect or hemisphere by group interaction in the WM tracts. No significant differences in the cortico-cortical association tracts and corticospinal tract were observed between any two groups. Recent studies suggest that the use of AxD and RD is more sensitive to WM damage than MD and FA. Therefore, we further analyzed group differences in AxD and RD measures (Table S1). Compared with controls, late aMCI subjects showed significant AxD and RD increases in the fornix, uncinate fasciculus, and parahippocampal cingulum. Early aMCI had significantly higher RD and AxD values of the fornix than that of controls. AxD and RD values of the uncinate fasciculus were higher in late aMCI than in early aMCI.

**Table 2:** Group comparisons of FA and MD measures in early aMCI, late aMCI, and controls.

	CN	Early aMCI	Late aMCI	F (p value)	Early aMCI vs. Late aMCI	Early aMCI vs. CN	Late aMCI vs. CN
	Mean (SD)	Mean (SD)	Mean (SD)				
Fx L FA	0.320 (0.027)	0.307 (0.023)	0.298 (0.032)	5.542 (0.005)	0.661	0.177	0.007*
Fx R FA	0.324 (0.028)	0.310(0.024)	0.305 (0.034)	4.552 (0.012)	1.000	0.127	0.030*
Fx L MD(mm^2^/s)	1.493 (0.142)	1.600 (0.112)	1.600 (0.123)	7.002 (0.001)	1.000	0.005*	0.030*
Fx R MD(mm^2^/s)	1.485 (0.142)	1.597 (0.120)	1.593 (0.128)	7.846 (0.001)	1.000	0.002*	0.027*
UF L MD(mm^2^/s)	0.871 (0.057)	0.884 (0.086)	0.931 (0.084)	7.172 (0.001)	0.017*	1.000	0.001**
UF R MD(mm^2^/s)	0.830 (0.047)	0.839 (0.058)	0.871 (0.052)	6.706 (0.001)	0.032*	1.000	0.001**
PHC L MD(mm^2^/s)	0.849 (0.046)	0.857 (0.048)	0.884 (0.074)	3.948 (0.021)	0.074	1.000	0.018*

Abbreviation: PHC, parahippocampal cingulum; Fx, fornix; UF, uncinate fasciculus; ILF, inferior longitudinal fasciculus; SLF, superior longitudinal fasciculus; CST, corticospinal tract. aMCI, amnestic mild cognitive impairment; CN, cognitively normal; L, left; R, right.

* Significance at p<0.05.

** Significance at P<0.01.

### The Relationship between WMHs, Hippocampal Volume and DTI Measures of WM Tracts

As shown in Table 3, in late aMCI subjects, left hippocampal volume was significantly correlated with left fornical FA, left uncinate fasciculus MD, and left parahippocampal MD, while right hippocampal volume did not correlate with ipsilateral DTI measures of these fiber tracts. In the early aMCI group, there were no associations between hippocampal volume and fornical DTI measures in either left or right hemisphere. None of DTI measures of WM tracts was associated with WMHs burden in either early aMCI or late aMCI.

**Table 3:** Results from the regression model of white matter tracts in early and late aMCI.

	Left Fx FA^a^	Right Fx FA^a^	Left Fx MD a	Right Fx MD^a^	Left UF MD^a^	Right UF MD^a^	Left PHC MD^a^	Left Fx MD^b^	Right Fx MD^b^
	Beta (p value)	Beta (p value)	Beta (p value)	Beta (p value)	Beta (p value)	Beta (p value)	Beta (p value)	Beta (p value)	Beta (p value)
Age	−0.156(0.473)	−0.202(0.379)	−0.058(0.801)	0.100(0.672)	0.021(0.917)	0.452(0.034)	0.164(0.453)	0.153(0.393)	0.147(0.431)
Sex	−0.117(0.576)	−0.219(0.326)	0.054(0.807)	0.011(0.962)	0.200(0.320)	0.235(0.236)	0.151(0.473)	−0212(0.302)	−0.301(0.163)
Education	−0.026(0.905)	−0.088(0.712)	0.199(0.405)	0.116(0.641)	0.196(0.361)	0.018(0.932)	−0.008(0.971)	0.113(0.605)	−0.028(0.904)
APOEε4	−0.105(0.621)	−0.198(0.377)	0.061(0.788)	0.059(0.798)	0.160(0.432)	0.095(0.629)	0.254(0.241)	−0.196(0.304)	−0.089(0.635)
WMHs	−0.052(0.794)	−0.188(0.368)	0.232(0.277)	0.317(0.152)	0.059(0.754)	−0.035(0.849)	0.061(0.757)	0.136(0.505)	0.128(0.546)
Left HV	0.594(0.008)	N/A	−0.413(0.066)	N/A	−0.569(0.007)	N/A	−0.545(0.013)	−0.299(0.136)	N/A
Right HV	N/A	0.393(0.078)	N/A	−0.364(0.144)	N/A	−2.632(0.017)	N/A	N/A	−0.062(0.758)

Abbreviation: PHC, parahippocampal cingulum; Fx, fornix; UF, uncinate fasciculus; WMHs, white matter hyperintensities; HV, hippocampal volume; FA, fractional anisotropy; MD, mean diffusivity.

a the late aMCI group.

b the early aMCI group.

### The Relationship between Episodic Memory and Anatomic Measures

Regression analysis showed that episodic memory performance was significantly associated with left parahippocampal cingulum MD, fornical FA bilaterally, fornical MD bilaterally, and left uncinate fasciculus, as well as left normalized hippocampal volumes. When all these anatomic structures were entered simultaneously into a single regression model, only left parahippocampal cingulum MD remained as a significant predictor of episodic memory performance (β = −0.173, t = −2.330, p = 0.021).

## Discussion

This study characterized WM microstructural injury and its relation to hippocampal atrophy at different stages of aMCI. We found that late aMCI individuals with severe memory deficits had significant microstructural WM abnormalities in the limbic WM tracts including the fornices bilaterally, the uncinate fasciculus bilaterally, and the left parahippocampal cingulum, compared with controls. In addition, hippocampal atrophy was found in late aMCI and correlated with DTI measures of limbic WM tracts. In contrast, early aMCI individuals with mild memory impairment demonstrated less severe WM tract damage with only bilateral fornices involved, while the hippocampus was still intact and did not associate with DTI measures of the fornix in early aMCI.

A previous DTI study using the region of interest method found that the fornix was more severely affected than the parahippocampal cingulum and left orbitofrontal WM in presymptomatic familial AD genetic mutation carriers who were destined to develop AD [41]. Similarly, APOE4 carriers, who are at increased genetic risk for the development of AD, also demonstrated significant FA reductions and MD increases of the fornix before the onset of clinical symptoms [42]. These results, along with our current findings, suggest that the fornix may be selectively more vulnerable than other limbic WM tracts in the early stage of prodromal AD.

Significant MD increases and unchanged FA were found in early aMCI, which is consistent with previous studies [20], [43] showing that diffusivity measures are more sensitive than FA alone in detecting early WM changes in AD. Since both RD and AxD values are increased proportionally in the fornix of early aMCI, FA measure which is determined by the ratio of these two diffusivity measures remains relatively unchanged. Recent AD animal model studies suggest that RD increase could reflect demyelination [44], [45], while AxD changes are related to axonal degeneration [46], [47]. Nonetheless, myelin and axonal loss could only be one possible neurobiological mechanism underlying DTI alterations of WM damage in MCI and AD. Other possible biophysical phenomena [48] such as fiber re-organization, changes in the space between intracellular compartments, and glial cell activation, can also be involved in AD-related WM changes, thereby complicating the explanation of DTI changes. In humans, although histopathological data could be used to determine what causes DTI alterations in WM, the time gap between in-vivo MRI scan and post-mortem examination makes interpretation of DTI changes challenging.

WM damage in AD and MCI is generally considered to be a consequence of Wallerian degeneration secondary to cell loss in the GM. Since the fornix has axonal connections with the cellular layer of the subiculum, which is a primary site of substantial neuronal loss within the hippocampus [49], one could speculate that hippocampal atrophy is very likely to be accompanied by secondary degeneration of the fornix. The present study found that hippocampal volume and microstructural integrity of the fornix were both reduced in the left hemisphere, and that smaller left hippocampus was highly correlated with lower FA and higher MD values of the left fornix in late aMCI individuals, which is in agreement with a recent study revealing a prominent association between hippocampal atrophy and fornical degradation in MCI patients [50]. Further analysis from the study by Lee et al [50] showed that there was a region-specific association between fornix injury and hippocampal thinning in the CA1 and subiculum areas, suggesting that both white and gray matter degenerative processes are not independent from each other over the course of AD development. Nonetheless, the present study showed that reduced WM integrity of the fornix was observed without either evidence of hippocampal atrophy or an association with hippocampal volumes in early aMCI. It is possible that MCI subjects in the study by Lee et al [50]are in more advanced stage than our early aMCI subjects, thus leading to the observed association between fornix degradation and hippocampal shrinkage, which is also evident in our late aMCI subjects but not in early aMCI. By contrast, the current study assessed the fornix-hippocampal association across a spectrum from early aMCI to late aMCI, thereby providing more insights into the evolution of sequential relationship between the two degenerative processes.

Two recent DTI studies found that fornical damage was independent of hippocampal atrophy in APOE4 carriers before the onset of cognitive impairment [42], [51]. Additionally, a previous study [20] investigating WM damage and its relationship to GM atrophy in AD and aMCI patients found that WM change in AD was strongly correlated with adjacent GM atrophy, whereas widespread WM alterations were found in aMCI in the absence of GM atrophy. Therefore, it was proposed that primary WM damage could precede GM abnormalities in the early stage of AD. In support of this claim, a post-mortem study [23] observed that WM atrophy unrelated to vascular pathology was present with no evidence of GM atrophy in preclinical AD. Taking these findings together, it is suggested that fornical degeneration in early aMCI is not simply a consequence of GM atrophy, but rather results from primary WM degeneration.

The etiology of this ‘primary’ WM degeneration in the fornix remains unclear. Small vessel disease is common in the elderly and may be the likely explanation for the WM changes. Since the frontal-subcortical circuits are the earliest and most vulnerable region to be affected by vascular pathology, the early clinical symptom of vascular disease is not memory decline, however. Although previous studies have reported ischaemia of the fornix, it only occurs in extremely rare circumstances [52], [53]. In addition, the present study showed that total WMHs load did not have an association with DTI measures of the fornix in early aMCI. Furthermore, from the WMHs distribution map in the early aMCI group, WMHs were found to be located in the periventricular areas and the corpus callosum, and no WMHs were observed in the fornix, further suggesting that ischaemic small vessel disease might not contribute to fornical damage in early aMCI. Nonetheless, we cannot rule out the possibility that subtle vascular disease may play a role in fornical damage in early aMCI, although the data did not appear to support this possibility.

We would like to consider the alternative possibility that the early change in WM seen in our aMCI subjects is primarily degenerative. The APOE4 allele, a well-established genetic risk factor for AD, has been linked to WM deficits in the brain regions selectively vulnerable to AD-related pathology [51], [54], [55]. In the current study, we found fornical degradation after regressing out the effect of the APOE4 allele, and DTI measures of the fornix did not associate with APOE4 allele, indicating that the presence of the APOE4 allele is not the main cause of fornical damage in the early course of disease process. The relationship between the APOE4 allele and AD risk has been shown to be age-dependent [56]. The APOE4 allele exerts its maximal effect on the risk of developing AD in patients with onset at ages between 65 and 70, which might partly explain why some studies did not observe any influence of the APOE4 allele on cognitive decline [57] and brain structural changes [58] in the older age group. Hence, the absence of the impact of the APOE4 allele on WM degradation would not be unexpected in our sample with a mean age close to 81. Furthermore, our current study used fiber tractography which is methodologically different from previous whole –brain DTI studies [51], [54], [55] demonstrating a deleterious effect of the APOE4 allele on the WM microstructure, which may account for some discrepancies in the findings.

Animal model studies [25], [59] have demonstrated that soluble amyloid-β peptides can cause demyelination of the WM prior to the presence of amyloid-β plaques and neurofibrillary tangles in the early presymptomatic stages of AD. Therefore, the toxic effect of amyloid-β peptides on the fornix should be considered as a possibility. Another study reported impaired axonal transport of the WM in AD mice, which preceded amyloid deposition by more than a year [24]. Abnormal axonal transport of the WM can result in axonal injury manifested as axonal swelling and spheroids, thereby severing as another possible mechanism underlying fornical degeneration in the early aMCI group. The fornix provides an important pathway for the delivery of nerve growth factor (NGF) from the hippocampus to the basal forebrain to maintain survival and functioning of the basal forebrain [60]. Meanwhile, anterograde axonal transport of cholinergic inputs from the basal forebrain to the hippocampus via the fornix facilitates production of brain-derived neurotrophic factor (BDNF) and its associated growth and proliferation of neurons in the hippocampus [61], [62]. Therefore, primary fornical abnormalities due to defective axonal transport might drive and promote downstream neuronal loss in the basal forebrain [63] and the hippocampus [64], which is supported by our finding that fornical degradation occurred with no evidence of hippocampal atrophy in the early aMCI group and loss of fornical WM integrity correlated with hippocampal atrophy in late aMCI individuals.

There were some limitations in the present study. First, the cross sectional nature of the analysis, even though the subjects were assessed over two waves, limited its ability to detect longitudinal changes of WM tracts. Second, it lacked amyloid imaging and histopathological data suggestive of AD pathology in aMCI subjects, thereby relying on clinical diagnosis only. Ongoing longitudinal follow-up will assess the proportion of aMCI subjects who will eventually convert to AD. Third, although the functional significance of limbic WM tracts in episodic memory was identified, the role of these fiber tracts in the encoding and retrieval components of episodic memory warrants further research. Fourth, the study did not evaluate other WM tracts critical for episodic memory, such as the mammillothalamic tract, which is too small to be reconstructed by the current DTI technique. Ultra-high resolution DTI is required to achieve a better understanding of WM tract damage in the early phase of prodromal AD. Despite these limitations, the present study demonstrated distinct patterns of WM tract abnormalities at different stages of aMCI, providing further insight into the temporal order of WM degeneration in prodromal AD.

## Supporting Information

Table S1 Group comparisons of AxD and RD measures in early aMCI, late aMCI, and controls. Abbreviation: PHC, parahippocampal cingulum; Fx, fornix; UF, uncinate fasciculus; ILF, inferior longitudinal fasciculus; SLF, superior longitudinal fasciculus; CST, corticospinal tract. aMCI, amnestic mild cognitive impairment; CN, cognitively normal; L, left; R, right.
(DOCX)
