# Supplementary material for: Microstructural White Matter Changes, Not Hippocampal Atrophy, Detect Early Amnestic Mild Cognitive Impairment
Source: PLoS One. 2013 Mar 14;8(3):e58887. doi: 10.1371/journal.pone.0058887 (PMC3597581; doi:10.1371/journal.pone.0058887)
Supplement: Table S1 — Group comparisons of AxD and RD measures in early aMCI, late aMCI, and controls. Abbreviation: PHC, parahippocampal cingulum; Fx, fornix; UF, uncinate fasciculus; ILF, inferior longitudinal fasciculus; SLF, superior longitudinal fasciculus; CST, corticospinal tract. aMCI, amnestic mild cognitive impairment; CN, cognitively normal; L, left; R, right. (DOCX) [file pone.0058887.s001.docx]

|  | CN | Early aMCI | Late aMCI | F ( p value ) | Early aMCI vs. Late aMCI | Early aMCI vs. CN | Late aMCI vs. CN |
| --- | --- | --- | --- | --- | --- | --- | --- |
|  | Mean (SD) | Mean (SD) | Mean (SD) |  |  |  |  |
| Fx L AxD | 1.977(0.140) | 2.080(0.110) | 2.062(0.094) | 6.035(0.003) | 1.000 | 0.004 | 0.063 |
| Fx R AxD | 1.969(0.141) | 2.081(0.119) | 2.061(0.099) | 7.242(0.001) | 1.000 | 0.001 | 0.039 |
| Fx L RD | 1.251(0.147) | 1.359(0.116) | 1.369(0.142) | 7.204(0.001) | 1.000 | 0.007 | 0.015 |
| Fx R RD | 1.242(0.146) | 1.355(0.124) | 1.359(0.146) | 7.752(0.001) | 1.000 | 0.004 | 0.016 |
| UF L AxD | 1.157(0.064) | 1.173(0.091) | 1.201(0.065) | 3.770(0.025) | 0.283 | 1.000 | 0.021 |
| UF R AxD  UF L RD  UF R RD | 1.124(0.056)  0.731(0.052)  0.677(0.049) | 1.126(0.070)  0.073(0.081)  0.687(0.051) | 1.162(0.053)  0.770(0.059)  0.711(0.051) | 3.556(0.030)  5.864(0.003)  5.506(0.005) | 0.049  0.055  0.163 | 1.000  1.000  0.722 | 0.040  0.002  0.004 |
| PHC L AxD  PHC R AxD  PHC L RD  PHC R RD | 1.151(0.056)  1.134(0.052)  0.699(0.049)  0.655(0.045) | 1.155(0.051)  1.135(0.051)  0.708(0.052)  0.659(0.058) | 1.186(0.087)  1.154(0.066)  0.733(0.070)  0.679(0.066) | 3.223(0.042)  0.946(0.390)  3.396(0.035)  1.702(0.185) | 0.093  0.534  0.136  0.392 | 1.000  1.000  1.000  1.000 | 0.044  0.785  0.031  0.215 |
|  |  |  |  |  |  |  |  |
|  |  |  |  |  |  |  |  |

Supplementary Table 1. Group comparisons of AxD and RD measures in early aMCI, late aMCI, and controls.

Abbreviation: PHC, parahippocampal cingulum; Fx, fornix; UF, uncinate fasciculus; ILF, inferior longitudinal fasciculus; SLF, superior longitudinal fasciculus; CST, corticospinal tract. aMCI, amnestic mild cognitive impairment; CN, cognitively normal; L, left; R, right.
